# Van der Waals interactions and the limits of isolated atom models at interfaces

**DOI:** 10.1038/ncomms11559

**Published:** 2016-05-13

**Authors:** Shigeki Kawai, Adam S. Foster, Torbjörn Björkman, Sylwia Nowakowska, Jonas Björk, Filippo Federici Canova, Lutz H. Gade, Thomas A. Jung, Ernst Meyer

**Affiliations:** 1 International Center for Materials Nanoarchitectonics, National Institute for Materials Science, 1-1, Namiki, Tsukuba, 305-0044 Ibaraki, Japan; 2 Department of Physics, University of Basel, Klingelbergstrasse 82, CH-4056 Basel, Switzerland; 3 Precursory Research for Embryonic Science and Technology (PRESTO), Japan Science and Technology Agency, 4-1-8, Honcho, Kawaguchi, 332-0012 Saitama, Japan; 4 COMP, Department of Applied Physics, Aalto University, PO Box 11100, FI-00076 Aalto, Finland; 5 Division of Electrical Engineering and Computer Science, Kanazawa University, Kanazawa 920-1192, Japan; 6 Physics/Department of Natural Sciences, Åbo Akademi University, 20500 Turku, Finland; 7 Department of Physics, Chemistry and Biology (IFM), Linköping University, Linköping 58183, Sweden; 8 Aalto Science Institute, Aalto University, PO Box 15500, FI-00076 Aalto, Finland; 9 Anorganisch-Chemisches Institut, Universität Heidelberg, Im Neuenheimer Feld 270, 69120 Heidelberg, Germany; 10 Laboratory for Micro- and Nanotechnology, Paul Scherrer Institute, CH-5232 Villigen, Switzerland

## Abstract

Van der Waals forces are among the weakest, yet most decisive interactions governing condensation and aggregation processes and the phase behaviour of atomic and molecular matter. Understanding the resulting structural motifs and patterns has become increasingly important in studies of the nanoscale regime. Here we measure the paradigmatic van der Waals interactions represented by the noble gas atom pairs Ar–Xe, Kr–Xe and Xe–Xe with a Xe-functionalized tip of an atomic force microscope at low temperature. Individual rare gas atoms were fixed at node sites of a surface-confined two-dimensional metal–organic framework. We found that the magnitude of the measured force increased with the atomic radius, yet detailed simulation by density functional theory revealed that the adsorption induced charge redistribution strengthened the van der Waals forces by a factor of up to two, thus demonstrating the limits of a purely atomic description of the interaction in these representative systems.

## Abstract (web summary)

Van der Waals forces are individually weak, but on scale can drive many nano- and macroscopic processes. Here, Kawai *et al*. directly measure the van der Waals interactions between noble gas atom pairs and show how this changes with atom size and surface adsorption.

## Graphical abstract

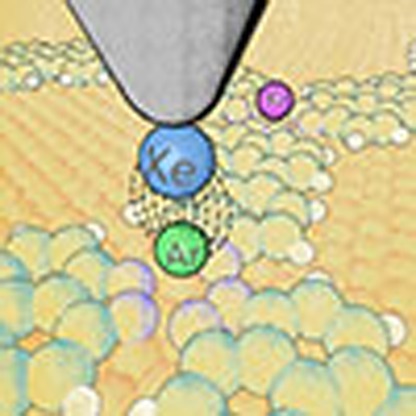

The van der Waals (dispersive) interaction^1^, arising from the temporal fluctuation of electronic charge inducing transient dipoles of variable strengths, is ubiquitous in nature and is of central importance for many physical and chemical phenomena. This interaction plays a key role in the condensation of atoms and molecules, friction and adhesion^2^, and is crucial for the adsorption of molecules on inert surfaces such as graphite^3^,^4^ and of *π*-conjugated molecules on metal surfaces^5^. While dispersion forces are inherently a quantum mechanical phenomenon, simple quasi-classical models have been applied very successfully^6^, and remain popular for many critical systems^7^,^8^. However, the advances of first-principles quantum methods, being applied to increasingly complex systems, have made it essential to include accurate van der Waals contributions and thus gain deeper insight into their physical consequences. Nonlocal correlations, responsible for dispersion interactions, are not included in the most commonly used functionals^9^, but more efficient approaches are constantly evolving^10^,^11^,^12^,^13^,^14^. Any new methods are usually rigorously tested against a set of known atomic structures or materials properties calculated using higher-order methods^15^, but as the systems studied increase in size and include large substrates and adsorbates, their capability is still limited by the current computational power. Therefore, experimental approaches to measure interactions in complex systems remain complementary to advances in quantum physical simulations. For example, this combination has proved successful in the direct probing of the van der Waals interaction between two Rydberg atoms^16^.

Atomic force microscopy (AFM) can directly measure the interaction between tip and sample down to the atomic scale via the frequency shift of an oscillating cantilever^17^, and by integrating the measured frequency shift in the *z*-direction, the force and potential can be extracted quantitatively^18^. This technique has been successfully used to discriminate embedded Si, Sn and Pb atoms in a Si(111) surface^19^ as well as In, Sn and Si atoms in a heterogeneous III–IV chain on a Si(100) surface^20^, and also to study the complex interplay between deformations and electronic states in Pt–Pt and Cu–Cu metal contacts^21^. Alongside such reactive systems, studies of weak interactions involving inert graphite^22^ and carbon nanotubes have been carried out^23^. Moreover, the role of van der Waals interactions between Au structures and aromatic hydrocarbons was explored by AFM tip-induced lifting of individual molecules^24^,^25^. However, direct force measurements of the atomic van der Waals potentials remain an unexplored topic to date. Rare gas atoms have long been providing a benchmark in van der Waals interactions due to their inert, monoatomic nature and the dominance of dispersion forces in their binding^26^.

In this work, we directly measure the van der Waals interaction between two adsorbed rare gas atoms. Individual Ar, Kr and Xe atoms were adsorbed and stabilized at a nodal site of a molecular network on Cu(111), and their interaction with a Xe-functionalized tip of AFM was measured at low temperature. We found that the magnitude of the van der Waals interaction scales with the size of the rare gas atom on the surface, that is, in order of Xe–Xe>Kr–Xe>Ar–Xe. A detailed theoretical simulation generally corroborated the experimental findings, and provided insight into the role of tip-induced atomic relaxations and charge transfer processes that cause deviations from a pure van der Waals interaction between two individual atoms.

## Results

### Rare gas atoms stabilized by 2D MOF

**Figure 1:**
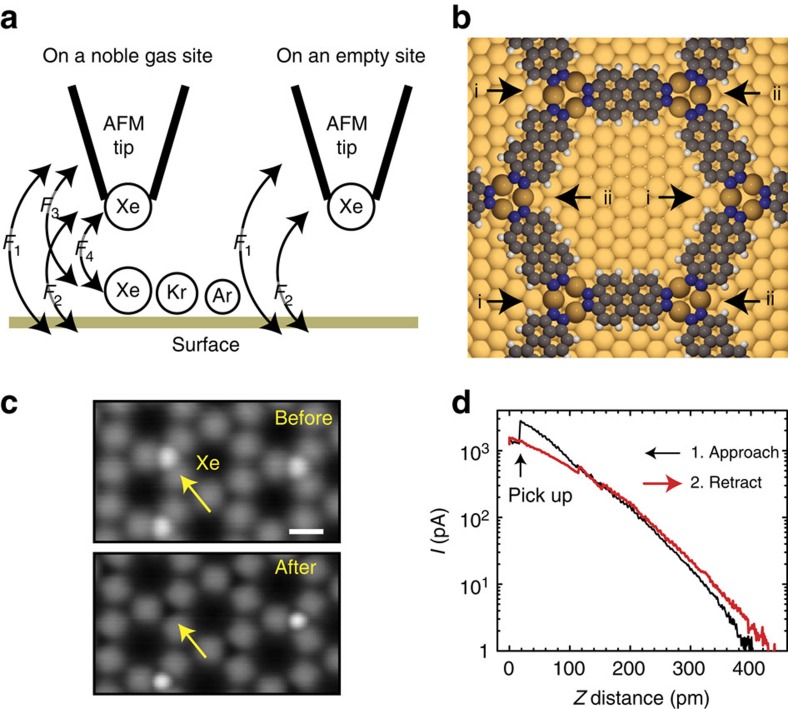
Rare gas atom stabilized by 2D MOF. (**a**) Schematic drawing of the AFM measurement set-up. (**b**) Chemical structure of Cu-coordinated molecular network on Cu(111); the two symmetry-inequivalent nodal sites are indicated as i and ii. (**c**) STM topographies before and after the vertical manipulation of a Xe atom. (**d**) Distance-dependent curve of the tunnelling current in the process. Measurement parameters: *I*=2 pA and *V*=200 mV in **c**, and *V*=2 mV in **d**. Scale bar, 1 nm.

In Fig. 1a, a schematic representation of the AFM experiment is depicted, with the interaction forces categorized into long-range and site-dependent short-range contributions. The system has four contributions; *F*_1_ the tip–sample surface interaction, *F*_2_ the Xe tip–sample surface interaction, *F*_3_ the tip–rare gas atom on surface interaction and *F*_4_ the Xe tip–rare gas atom on surface interaction. The *F*_4_ contribution corresponds to a direct component between the two atoms, which at long range is given by the van der Waals interaction. To extract the latter components, reference measurements were carried out at an equivalent atomic site in absence of a surface adsorbed rare gas atom (denoted ‘empty site' in Fig. 1a). By subtracting the two *z*-distance-dependent curves, the interaction force of *F*_3_+*F*_4_ could be determined directly. *F*_1_–*F*_4_ have different power laws and origins of the *Z* distance. Among them, *F*_4_ has the largest power law and the smallest *Z* distance for a given tip–sample distance. We first attempted to probe the force of Xe adsorbed on a flat Cu(111) surface (Supplementary Fig. 1)^27^. However, the high mobility of the rare gas atoms on the metal surface hampered a reliable measurement. In fact, the diffusion barrier was so low that the rare gas atom was often accidentally moved in the lateral direction by the tip–sample interaction (Supplementary Fig. 2).

To overcome these problems, we employed a surface-confined two-dimensional metal–organic framework (2D MOF) as an anchor net for the inert rare gas atoms. We formed the 2D MOF^28^ by depositing the perylene derivative 4,9-diaminoperylene-quinone-3,10-diimine (DPDI) on a clean Cu(111) surface, and then annealing the sample at ∼300 °C. Recent work has shown that a series of dehydrogenation reactions and subsequent coordination of the molecule's nitrogen atoms to Cu adatoms results in the honeycomb structure depicted in Fig. 1b (ref. 29). As a result of the adsorption geometry of adatoms and molecules on the surface, the 2D MOF has two kinds of coordination motifs, indicated as i and ii (see also Supplementary Fig. 3), each comprising three Cu adatoms and which are referred to as nodes in the honeycomb network. Figure 1c shows that Xe atoms, dosed onto the cold sample (<8 K), adsorb predominantly on node sites. By setting a clean Cu tip 400 pm closer to the Xe site than the imaging distance^30^, the tip was reliably functionalized by the Xe atom (Fig. 1d).

### Atomic scale imaging of the 2D MOF

**Figure 2:**
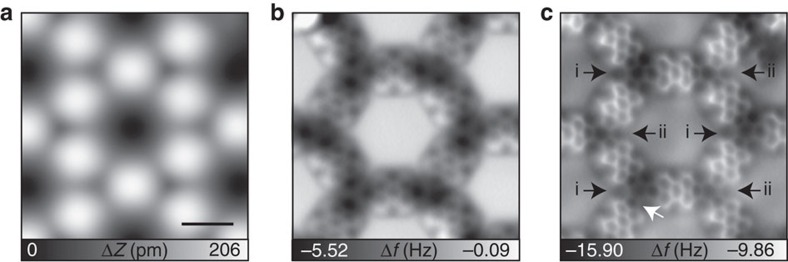
Atomic scale observation. (**a**) STM topography and (**b**) AFM images taken with a Xe-functionalized tip and (**c**) a CO-functionalized tip. The two symmetry-inequivalent nodal sites are indicated as i and ii; the five-membered C_3_N_2_ of the 3deh-DPDI exo-ligands are indicated by a white arrow. Measurement parameters: *I*=2 pA and *V*=−200 mV in **a**, and *A*=60 pm and *V*=0 mV in **b**,**c**. Scale bar, 1 nm.

Figure 2a displays the scanning tunnelling microscopy (STM) topography, taken with a Xe-functionalized tip. Each molecule appears as a featureless ball-like shape, reflecting the molecular electronic structure, as established in previous measurements^28^. To obtain more details of the molecular structure, we took advantage of the capabilities of AFM with functionalized tips^31^,^32^,^33^,^34^,^35^,^36^. In Fig. 2b, the frequency shift map taken at a constant height is shown, in which the chemical structure at the centre of the perylene core becomes apparent but the molecular units remain blurred at the nodes. Since the measurement was performed in the repulsive interaction region, the darker area (more negative frequency shift) corresponds to a larger tip–sample separation, that is, the nodes are closer to the Cu substrate and the nitrogen atoms are thus not resolved.

To enhance the resolution, we switched to a CO-functionalized tip, which is known to improve resolution compared with a Xe tip due to its smaller atomic radius and higher flexibility^37^. Figure 2c shows the frequency shift image, in which the chemical bonds in the perylene derivative now appear clearly. The carbon rings in perylene are apparently elongated in the direction of the short molecular axis due to the tilting effect of the CO molecule on the tip apex^31^. Even the C_3_N_2_ pentagonal rings of the triply dehydrogenated (3deh)-DPDI exo-ligands at the nodes are resolved as indicated by a white arrow. Since the nitrogen atoms are coordinated with Cu adatoms on the surface, the end groups of the molecule are closer to the Cu substrate than the perylene core, in agreement with X-ray standing wave measurements and theoretical calculations of the system^29^. The Cu adatoms coordinated to the nitrogen atoms are not visible in the images, although they have been identified indirectly by an STM fingerprint of the metal–organic coordination at particular bias voltages^29^. Closer inspection reveals that the two distinct nodal positions of the honeycomb network can be resolved by the adsorption of Xe atoms, which appear in STM images with different apparent sizes (Supplementary Fig. 4). This experimental result unambiguously supports the previous theoretical calculations and experiments^29^.

### Van der Waals force detection

**Figure 3:**
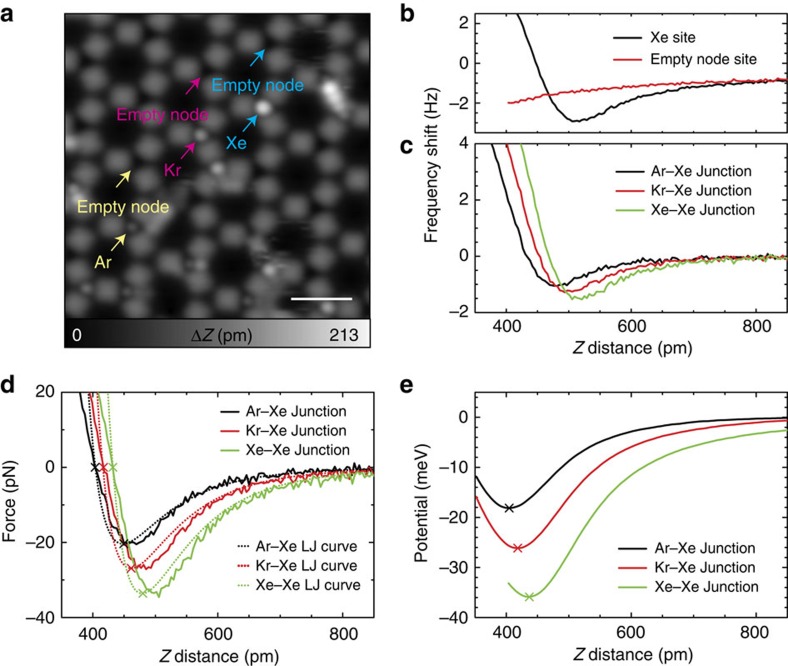
van der Waals force detection. (**a**) STM topography of the Cu-coordinated 3deh-DPDI network after measuring interaction curves above Ar, Kr and Xe atoms. (**b**) Frequency shift curves taken at the equivalent node sites with and without a Xe atom, measured with a Xe-functionalized tip. (**c**) Subtracted distance-dependence curves of the frequency shift for Ar–Xe, Kr–Xe and Xe–Xe junctions. (**d**) *F*_4_ interaction forces and Lennard-Jones fits for each system, extracted from the frequency shift signal and (**e**) the equivalent potential energy curves. Measurement parameters: *V*_tip_=500 mV and *I*=4 pA in **a**, and *A*=38 pm, *f*=23,063 Hz and *Q*=52,044, and *V*=1 mV in **b**,**c**. Scale bar, 2 nm.

To prepare the test bench for interatomic van der Waals force measurements, Ar, Kr and Xe gases were deposited on the substrate with the Cu-coordinated 3deh–DPDI network. Figure 3a shows a STM topography, in which the node sites are partially filled by Ar, Kr and Xe atoms. The observed apparent sizes of the rare gas atoms relate to the van der Waals radii (*R*_Ar_=188 pm, *R*_Kr_=202 pm and *R*_Xe_=216 pm), though they do not scale quantitatively since, for instance, the Ar atom is observed as about half the size of the Xe atom. As indicated above, two kinds of node sites can be differentiated via the apparent sizes of the same rare gas (Supplementary Fig. 4). We also observed that some atoms adsorbed to the pore sites and they could be easily manipulated by a scanning tip^38^, whereas ones at the node sites were found to be highly stable.

In Fig. 3b, distance-dependent curves of the frequency shift are displayed, which were measured on the Xe-filled and -empty nodal sites by the Xe-functionalized tip. The reference site (empty node) was selected at the nearest symmetry-equivalent node, as indicated by the arrows in Fig. 3a. Before the distance-dependent measurement, the *z*-feedback was deactivated so that the relative *z*-distances of two curves were consistent. However, in the experiment, the absolute *z*-distance from the surface was undefined, and the origin of the *z*-distance therefore set *a posteriori* by equating the equilibrium force distance to the sum of the van der Waals radii (Ar–Xe=404 pm, Kr–Xe=418 pm and Xe–Xe=432 pm).

On the Xe-filled node site, a strong site-dependent interaction is detected while no significant site-dependent force was observed on the corresponding empty node site. Above the empty site, the tip–sample separation is so large that only the macroscopic attractive interaction is present and the tip does not approach close enough to experience short-range interactions in the plotted tip–sample separation range (equivalent to *F*_1_+*F*_2_, see Fig. 1a). By subtracting two curves measured at the Xe-filled and -empty node sites, we obtain the *F*_3_+*F*_4_ distance-dependent curve. Since the origin of the *z*-axis in the *F*_3_ distance-dependent curve is shifted by the size of Xe atom, its contribution is smaller than that of *F*_4_. Further, the tip was ultra-sharpened by focused ion beam milling, which also contributed to the reduction of *F*_3_ (Supplementary Fig. 5), giving a relation of *F*_2_>*F*_3_. Thus, the maximum contribution of *F*_3_ can be estimated via *F*_2_ (Supplementary Fig. 6). We estimated the contribution of *F*_2_ via fitting the different power laws in the *z*-distance-dependent frequency shifts on the empty node site and consequently found that the contribution of *F*_3_ is <5% of *F*_4_. Therefore, by subtracting two curves measured at the Xe-filled and -empty node sites, we could estimate the interatomic van der Waals interaction (*F*_4_).

To compare a magnitude of *F*_4_ for Ar–Xe, Kr–Xe and Xe–Xe atom pairs, the *z*-distance-dependent frequency shifts were measured with the same Xe-functionalized tip as shown in Fig. 3c. To obtain a more quantitative physical value, the force was extracted via the measured frequency shift with the aid of a numerical algorithm (Fig. 3d)^18^. The magnitude of the maximum attractive force between two noble gas atoms was found to be <35 pN, which is much smaller than what has been found in previous measurements on semiconductors and metals^19^,^20^,^21^. By integrating the force in the *z*-direction, the potential between Ar–Xe, Kr–Xe and Xe–Xe were obtained as shown in Fig. 3e.

In a first attempt to relate these curves to a simple physical model, the corresponding force versus distance relations calculated from Lennard-Jones models^6^, where the equilibrium force distance and the depth of the force were adjusted to the experimental data, were determined and are shown with broken lines in Fig. 3d. From these, the depths of the potential wells were derived as *ɛ*_Ar–Xe_=19 meV, *ɛ*_Kr–Xe_=26 meV and *ɛ*_Xe–Xe_=34 meV, which are in reasonable agreement with the depths of the potential curves directly extracted via the measured frequency shift, that is, 18.1 meV for a Ar–Xe dimer, 26.1 meV for a Kr–Xe dimer and 35.9 meV for a Xe–Xe dimer. However, the shapes of the fitted force curves deviate from the measured ones, indicating that the simple and rigid Lennard-Jones fitting was not suitable for a precise representation of the experimental results.

### Theoretical simulations

**Figure 4:**
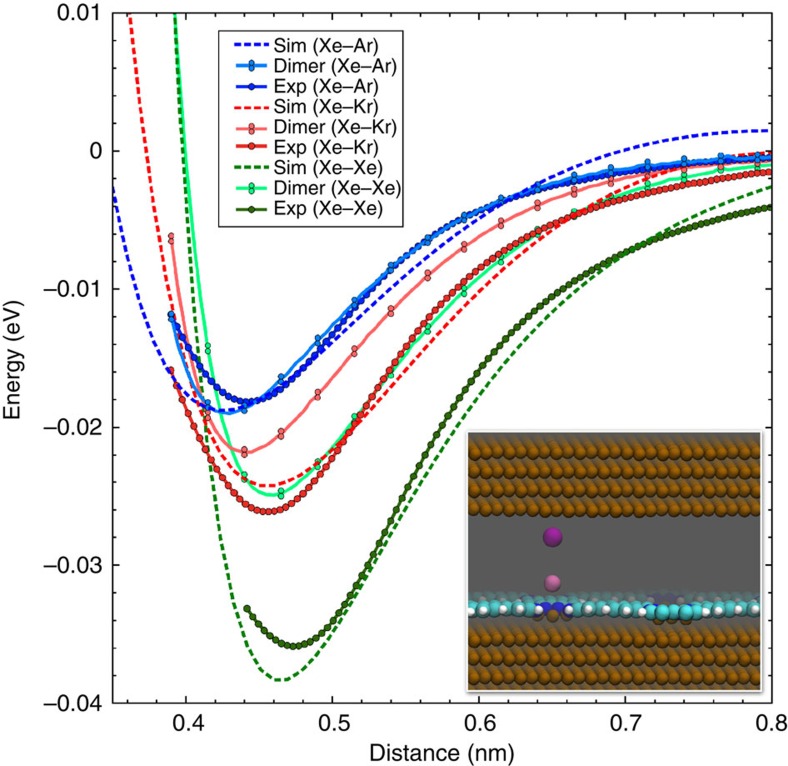
Theoretical calculation. Comparison of simulated tip–surface and dimer potential energy curves with experimentally derived curves (the same experimental data as in Fig. 3e) for each rare gas atom pair. The *z*-position of the experimental curves has been normalized with respect to the simulated Xe–Kr tip–surface curve. The curves were fitted with polynomial functions. Inset: atomic snapshot of the simulated tip–surface system.

To demonstrate the dimer-specific relaxation of the adatom positions and the conclusions to be drawn on the interatomic potentials in detail, we used first-principles simulations to calculate potential energy curves using a Xe on copper tip (the calculated system is shown in the inset of Fig. 4) above the three different rare gas atoms adsorbed on the node and empty sites. As in the experiments, the curve above the empty site was subtracted from the other curves to remove background interactions (Supplementary Fig. 7). Figure 4 shows a comparison of the experimental short-range potential energy curves with the simulated tip–surface and bare dimer curves. For Xe–Ar, all three curves are reasonably close to each other. In the Xe–Kr curves, the simulated and measured tip–surface curves are also in very good agreement, but both significantly overestimate the calculated dimer potential energy. Finally, the Xe–Xe curves show the largest deviation from the dimer potential, with the simulated and measured tip–surface curves in reasonable agreement, but with a minimum potential energy which is almost double that of the dimer.

To establish possible explanations for the differences from the pure dimer potential energies, we analysed the atomic positions and electron density during the simulated tip–surface approaches. We found that major tip-induced displacements of the noble gas atoms occur at a separation of <0.5 nm, in the region of strongest interaction—in particular as the repulsive part of the potential is entered. In this region, the Xe-tip and atom–surface network bond lengths gradually reduce (up to ∼0.03–0.04 nm at a separation of 0.3 nm) as the tip approaches. There is no systematic difference between Ar, Kr and Xe in this regard, although the displacements will have different relative energetic costs in each system. Since the energy cost of these relaxations is very specific to the approach site, they cannot be cancelled by subtraction of the empty site potential curve.

There is also a clear systematic difference when comparing electron transfer in the three systems. As in the binding energy, and clearly linked to it, the magnitude of electron transfer between the investigated atoms and the surface network/metal tip correspondingly scales as Xe>Kr>Ar. Specifically, at a separation of 0.5 nm for each system, the Bader charge (integrating the electron density) on the surface noble atom is 0.01/0.03/0.09 *e* less than in the equivalent dimer configuration for Ar/Kr/Xe, respectively. A similar difference (0.03 *e*) for Xe on the tip is also observed for each system. Analysis of the nature of the electron density transfer (Supplementary Fig. 8) shows that this is dominated by transfer from the rare gas atom into bonding states between the same atom and the tip or surface, and to copper and/or nitrogen if resting on the nodes of the network. Although this is mitigated by subtraction of the empty site reference potential (which has charge transfer contributions) to some degree, the electron transfer is significantly smaller in the region of the strongest interaction for the empty site system.

The observed difference between pure van der Waals binding and our results on Xe to Ar/Kr/Xe dimers, along with the induced relaxation, certainly plays a significant role in the differences between the experimental and simulated tip–surface energy potentials and the pure atom–dimer curves. As the binding strength and electron transfer increases, so does the deviation, reducing the validity of subtraction of the empty site reference curve, such that ultimately the measured Xe–Xe interaction is almost double the bare dimer interaction, while this discrepancy is not observed for the Xe–Ar potential. Using an Ar-functionalized tip would mitigate the effect, but attempts to this end gave rise to severe issues with tip stability that precluded reliable measurements.

Despite these difficulties to correctly describe the depth and position of the potential minimum based on a simple dimer picture, the asymptotic behaviour of the interaction at large distances is very similar to the asymptotics of the dimer curves. For dimers, or more generally any two point-like polarizable objects, the interaction potential asymptotically at long range has a leading term from the van der Waals interaction, which takes the form *C*_6_/*r*^6^ as a function of the distance, *r*. The interaction strength is determined on the dynamical polarizabilities of the individual atoms^39^. The curves of Xe–Ar and Xe–Kr in this region fit very well to such a limiting −*C*_6_/*r*^*−*6^ behaviour expected for the interaction between two rare gas atoms (Supplementary Fig. 9), although the *C*_6_ coefficients obtained in this way are very large, 2.5 and 3.4 times higher than the free-ion cases (Table 1). Since the tail of the interaction extends beyond a distance at which direct charge transfer between tip and substrate is to be expected, the most natural explanation of this enhancement is the notion that the change in polarizability mostly stems from the rare atom adsorbed on the surface and its interaction with the substrate. For the Xe–Xe interaction, the optimal asymptotic form is *r*^−5^. For consistency of comparison, Table 1 gives the value of the *C*_6_ coefficient from a fit to the non-optimal *r*^−6^ form, which also in this case is much larger than the free atom value, reflecting the much stronger binding. The deviation from the pure *r*^−6^ form indicates that the interaction has contributions also from sources other than the van der Waals interaction that stems from the polarizability of the objects. Our calculations show the formation of weak bond dipoles both at the tip and the substrate through charge transfer. Two constant dipoles should result in an interaction decaying as *r*^−3^, which is not seen for Ar and Kr, so it seems as an unlikely contribution also for Xe. Furthermore, the interaction between dipoles oriented as in our calculations should repel, giving a positive energy contribution and thus decrease the interaction, which is the opposite of what we see. Performing a fit to a sum −*C*_6_*r*^−6^−*C*_*N*_*r*^−*N*^ functions yields near-perfect fits for any *N*=2, 3, 4 and 5, and so is not helpful for disentangling these interactions. However, Xe is well known to be more prone to covalent interaction than both Kr and Ar, which may result in either electrostatic interaction due to the larger charge transfer to the substrate or a stronger covalent bond for the Xe–Xe dimer. Over the measured interval, we are not able to clearly distinguish these two scenarios for the Xe–Xe interaction.

## Discussion

We present a quantitative force measurement of van der Waals forces in the atomic-scale contacts of Argon–Xenon, Krypton–Xenon and Xenon–Xenon atoms. These rare gas atoms were stabilized in the nodal sites of a metal-coordinated two-dimensional molecular network, and the probing AFM tip was prepared by picking up a Xe atom from the nodal site. Systematic force measurements established the depths of the potential curves for Xe–Ar, Xe–Kr and Xe–Xe at 18.1, 26.1 and 35.9 meV, respectively, which is in agreement with the expected trend. The tails of the interactions of Xe–Ar and Xe–Kr were found to display van der Waals *r*^−6^ limiting behaviour; however, the effective strength of the interaction was found to depend on the influence of the tip and the substrate on the atomic polarizability, leading to a deviation from the behaviour of isolated noble gas atom. For Xe–Xe interaction, we find a deviation from the van der Waals power law, which we interpret as an effect of stronger covalent interaction between the Xe atoms. This approach offers a systematic way to characterize the interaction between any atomic and molecular species that can be fixed at a solid surface of a solid or be templated by a surface framework, and has the sensitivity to measure even the weakest forces. The combination with theory has allowed for a detailed analysis of the interaction components, clearly highlighting the role of ‘classical' van der Waals interactions while identifying the limits of an isolated atom model.

## Methods

### Experimental measurement

All experiments were performed with Omicron STM/AFM with a qPlus configuration^40^, operating at 4.8 K in ultrahigh vacuum (UHV). A clean Cu(111) surface was *in situ* prepared by repeated cycles of standard sputtering and annealing. The W tip of a tuning fork sensor was *ex situ* sharpened by focused ion beam milling technique and was then *in situ* covered with Cu atoms by contacting to the sample surface. Perylene derivative, 4,9-diaminoperylene-quinone-3,10-diimine (DPDI) molecules^41^ were deposited on the surface from a Knudsen cell, resistively heated at 200 °C. By annealing at ∼300 °C, the molecular network was formed^28^. The resonance frequency of the self-oscillating qPlus sensor was detected by a digital lock-in amplifier (Nanonis: OC4 and Zurich Instruments: HF2LI and PLL). Xe, Kr and Ar gases were sequentially dosed via an UHV leak valve while the sample stayed in a cold microscope. In STM mode, the tip was biased, while the sample was electronically grounded. The topographic images were taken in a constant current mode. In AFM mode, the tip apex was terminated by a CO or Xe molecule and all images were taken at a constant height mode.

### Theoretical simulation

All first-principles calculations in this work were performed using the periodic plane-wave basis Vienna ab initio simulation package (VASP) code^42^,^43^ implementing the spin-polarized density functional theory. To accurately include van der Waals interactions in this system, we used the optB86B+vdW-DF functional^44^,^45^,^46^, selected based on benchmarks (Supplementary Fig. 10) and previous work showing that it provides a sufficiently accurate description for all subsystems involved in the measurement. Projected augmented wave potentials were used to describe the core electrons^47^, with a kinetic energy cutoff of 550 eV (with PREC=accurate). Systematic *k*-point convergence was checked for all systems, with sampling chosen according to the system size and the gamma point being used for the largest systems. This approach converged the total energy of all the systems to the order of meV. The properties of the bulk and surface of Cu, each noble atom dimer pair and their adsorption on copper, were carefully checked within this methodology, and excellent agreement was achieved with experiments. Bader charge analysis was used to estimate charge transfer in the simulations^48^.

The geometry of the Cu-coordinated 3deh-DPDI network was taken from previous studies^29^. In general, the interaction strength with the network scaled as Xe>Kr>Ar (see Supplementary Table 1), which is consistent with the experiments and expected from previous studies^49^. To represent the tip, we used an equivalent (111) Cu slab to that under the DPDI network (see inset in Fig. 4), with the Xe tip atom adsorbed in its equilibrium position—since the tip is directly contacted to the copper surface in experiments, this seems a plausible model (good agreement with experimental studies of Xe adsorption on Cu (111) was achieved^50^—see Supplementary Table 1). Other, smaller copper tip models were considered, but were either very unstable or showed poorer agreement with the experimental curves. The interaction parameters were obtained by fitting the single parameter expression *C*_*N*_/*r*^*N*^ to the asymptotic tail of the experimental data, defined as starting just above the inflection point of the curve. The appropriate power, *N*, was selected based on the quality of the fits as measured by the correlation coefficients.

## Additional information

**How to cite this article:** Kawai, S. *et al*. Van der Waals interactions and the limits of isolated atom models at interfaces. *Nat. Commun.* 7:11559 doi: 10.1038/ncomms11559 (2016).

## Supplementary Material

Supplementary Information Supplementary Figures 1-10, Supplementary Table 1 and Supplementary References

## Figures and Tables

**Table 1:** Comparison of *C*_6_ coefficients fitted to AFM measurements and theoretical reference^39^.

Dimer	*C*_6_ (atomic units)		Ratio
	**AFM measured**	**Reference**	
Xe–Ar	341	135.0	2.5
Xe–Kr	662	192.9	3.4
Xe–Xe	1,267*	287.5	4.4

AMF, atomic force microscopy.

Note that the value for Xe–Xe, marked with an asterisk, does not correspond to the optimal fit.
