# Supplementary material for: Van der Waals interactions and the limits of isolated atom models at interfaces
Source: Nat Commun. 2016 May 13;7:11559. doi: 10.1038/ncomms11559 (PMC4869171; doi:10.1038/ncomms11559)
Supplement: Supplementary Information — Supplementary Figures 1-10, Supplementary Table 1 and Supplementary References [file ncomms11559-s1.pdf]

Supplementary Information:

Supplementary Figures

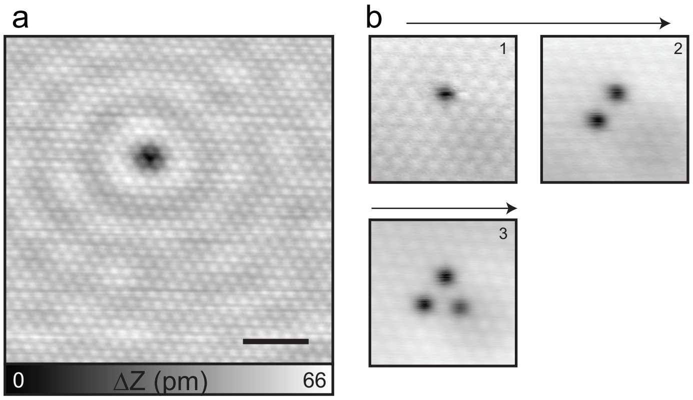

Supplementary Figure 1. **Xe adsorbed on a Cu(111) surface.** (**a**) Scanning tunnelling microscopy (STM) topography of Xe layer adsorbed on Cu(111). Xe gas was introduced via an ultra-high vacuum (UHV) leak valve (30 Langmuir) while the sample stayed in the cold microscope. It is known that the dark hole relates to the CO atom underneath the Xe atoms. The modified dispersion of the Cu surface state by Xe atom is also observed. [1]. (**b**) A series of STM topographies during the tip-induced vertical manipulation. Three Xe atoms were successfully extracted by a local probe with a method described in the main text. The vacancy sites can in principle be used as reference for the force measurement, yet the noise at the vacancy sites indicates random and infrequent movements of the Xe atoms. For this instability, a reliable force measurement was hardly performed. Further, since Kr and Ar have a lower diffusion barrier (boiling point; 87.4 K for Ar, 112.5 K for Kr, and 166.6 K fo Xe), they would lead even higher rates of the lateral diffusion. Measurement parameter: $V = 10$ mV and $I = 10$ pA in **a** and $V = 200$ mV and $I = 20$ pA in **b**. Scale bar, 2 nm.

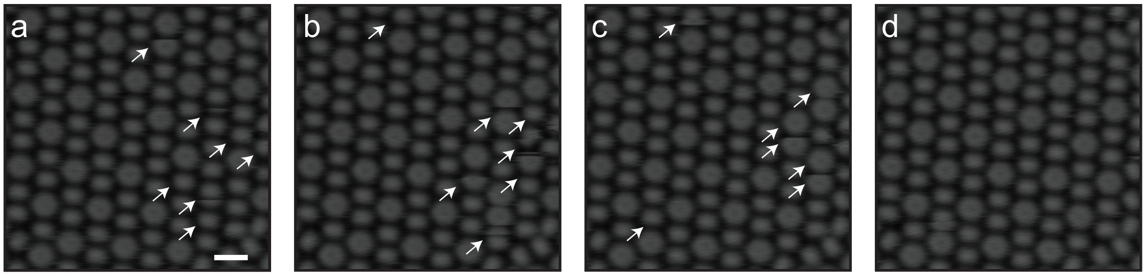

Supplementary Figure 2. **Diffusion of Xe on a Cu(111) surface.** (**a**)-(**d**) A series of STM topographies of Xe atoms adsorbed on Cu(111). To investigate the diffusibility of the Xe atoms, we prepared the submonolayer Xe film. After the measurement shown in Fig. S1, the whole microscope and cryostat were once heated up (55 K at the sample) and were cool down to 4.8 K. In this process, the cold Xe atoms were once desorbed from the wall of the cryostat and then adsorbed on the sample during cooling. The low kinetic energy, compared to that of the room-temperature Xe atoms introduced via the UHV leak valve, leads to the cluster like conformation. A similar measurement has been previously done with Ar atoms on Ag(111) by Heyde *et al.*, [2] In our case, dimers and hexamers of Xe atoms co-existed. Although the tip-sample separation was set large, the diffusion of the atoms was frequently observed as indicated by arrows. Note that the STM junction is several hundreds pm larger than the point where the most attractive force is expected. This result indicates the difficulty to measure the force curve over an isolated single Xe atom on Cu(111) since the tip-sample interaction induces the lateral diffusion of the Xe atom. Measurement parameter: $V = 900$ mV and $I = 10$ pA. Scale bar, 1 nm.

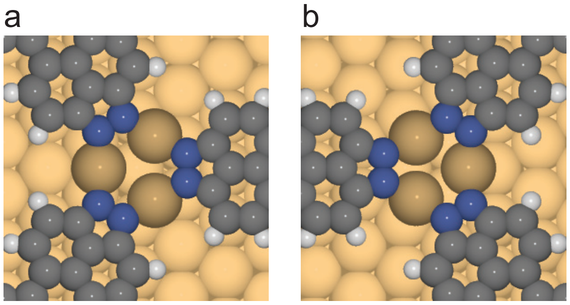

Supplementary Figure 3. **Detailed atomic structures of the node sites of the Cu-coordinated 3deh-DPDI network on Cu(111).** (**a**) Chemical structure of the type-1 node site in the network. [3] The centre of the node site locates above the surface Cu atom. (**b**) Chemical structure of the type-2 node site in the network. The centre of the node site locates above a hollow site of the Cu(111) surface.

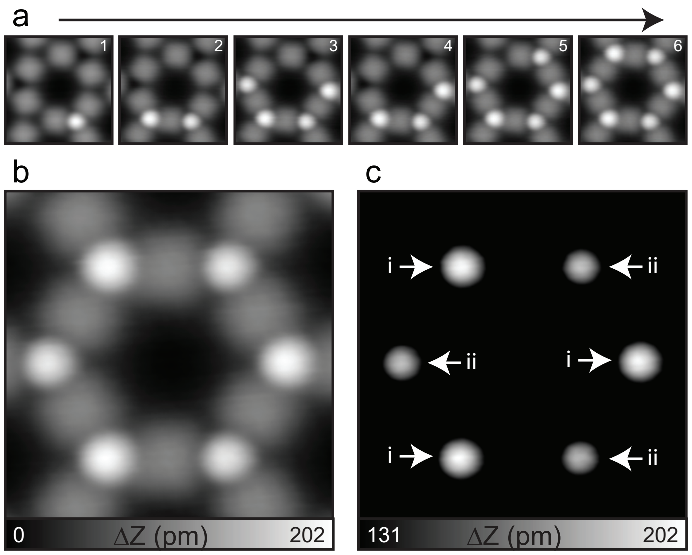

Supplementary Figure 4. **Different size of Xe atoms on different node sites.** (**a**) A series of STM topographies during atom-by-atom manipulation. Individual Xe atoms were repositioned to the node sites of the Cu-coordinated 3deh-DPDI network by a method described in a previous literature [4]. (**b**) STM topography, showing that the six node sites are covered by six Xe atoms after the manipulation. (**c**) Corresponding STM topography with a narrowed contrast for a better comparison of the Xe atom sizes. Two different sizes of Xe atom, located on i and ii three-fold node sites, are clearly seen. The difference of the apparent height was 20 pm. This measurement clearly indicates that the two different node structures induce the different charge transfer to the substrate. Measurement parameter: $V = 500$ mV and $I = 20$ pA.

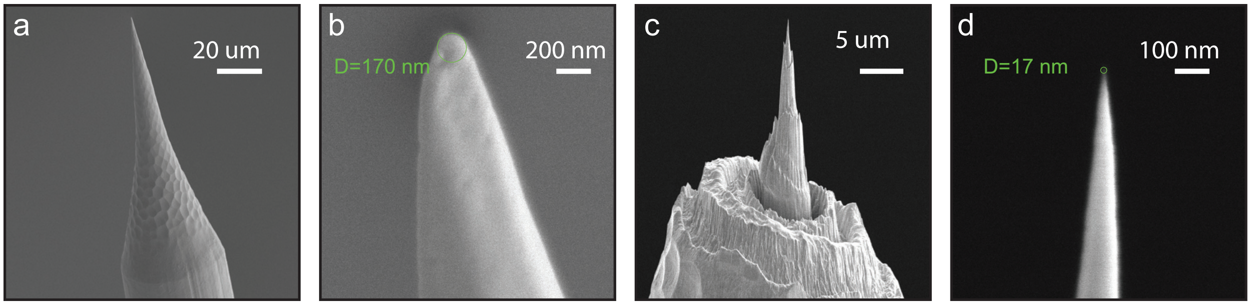

Supplementary Figure 5. **SEM images of the ultra-sharp tip obtained by focused ion beam (FIB).** (**a**) Scanning electron microscopy (SEM) image of an electro-chemically etched tungsten tip for STM and AFM. (**b**) Magnified SEM image. The measured tip diameter is approximately 170 nm. (**c**) SEM image of the ultra-sharpen tip obtained by FIB milling. (**d**) Magnified SEM image. The measured tip diameter is approximately 17 nm. The milling procedure can be found in the literature. [5] The reduced tip diameter by a factor of 10 leads to the drastic reduction of long-range tip-sample interaction in the force measurement (F_1 and F_3 in Fig. 1 of the main text).

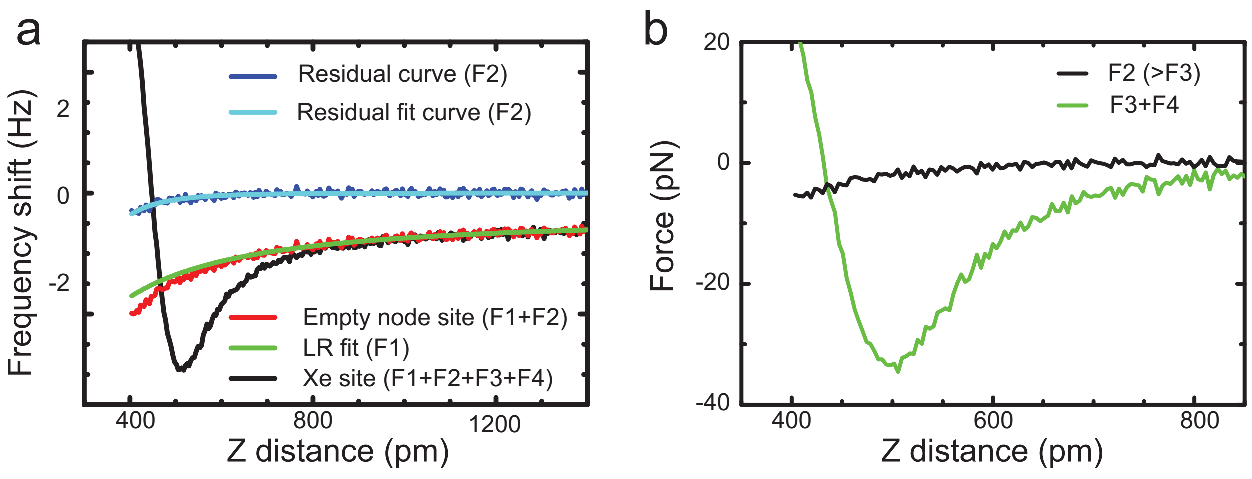

Supplementary Figure 6. **Contribution of F_3 estimated via fitting.** In this experiment, the tip was sharpened by the focused ion beam such that F_3 would be smaller than F_2. While the contribution of F_3 cannot be directly extracted, the magnitude can be estimated via F_2. (**a**) The distance dependent frequency shift, measured on the empty node site, contains the contributions of F_1 and F_2. Since F_1 is the long-ranged (LR) interaction, it can be extracted by fitting the tail of the distance dependent frequency shift on the empty node site with a function of $\Delta f(z) = -a \times z^b + c$, where z is the tip-sample Z distance, a the intensity, b the power law, and c is the offset frequency (0.4 Hz) , which comes from the error to set zero frequency shift in the experiment. We obtained the b value of -1.47 , which is smaller than the van der Waals interaction between surface and tip ($b \approx -3$). Thus, the main contribution seems to be the electrostatic interaction, arising from the different contact potential between tip and sample. Then, the residual curve was obtained by subtracting the empty node site curve and the LR fit curve. In this way, the contribution of F_2 in the frequency shift was obtained. Further, we fit the residual frequency shift curve with a function of $\Delta f(z) = -d \times z^e$, where d is the intensity and e is the power law. The obtained value of e is -5.40, which is close to the value for the van der Waals interaction between the rare gas atom and the tip (F_2). (**b**) Extracted F_2, obtained by integrating the residual frequency shift. We found that F_2 is approximately 2 pN at Z = 0.5 nm, where the maximum force value of $F_3 + F_4$ was detected ($\approx$ 6% in total). F_3 is smaller than F_2 due to the smaller volume in the ultra-sharp tip (on the macroscopic scale) than the flat Cu(111) crystal. Therefore, the contribution of F_3 is negligible in the subtracted curves measured at the Xe-filled and empty node sites.

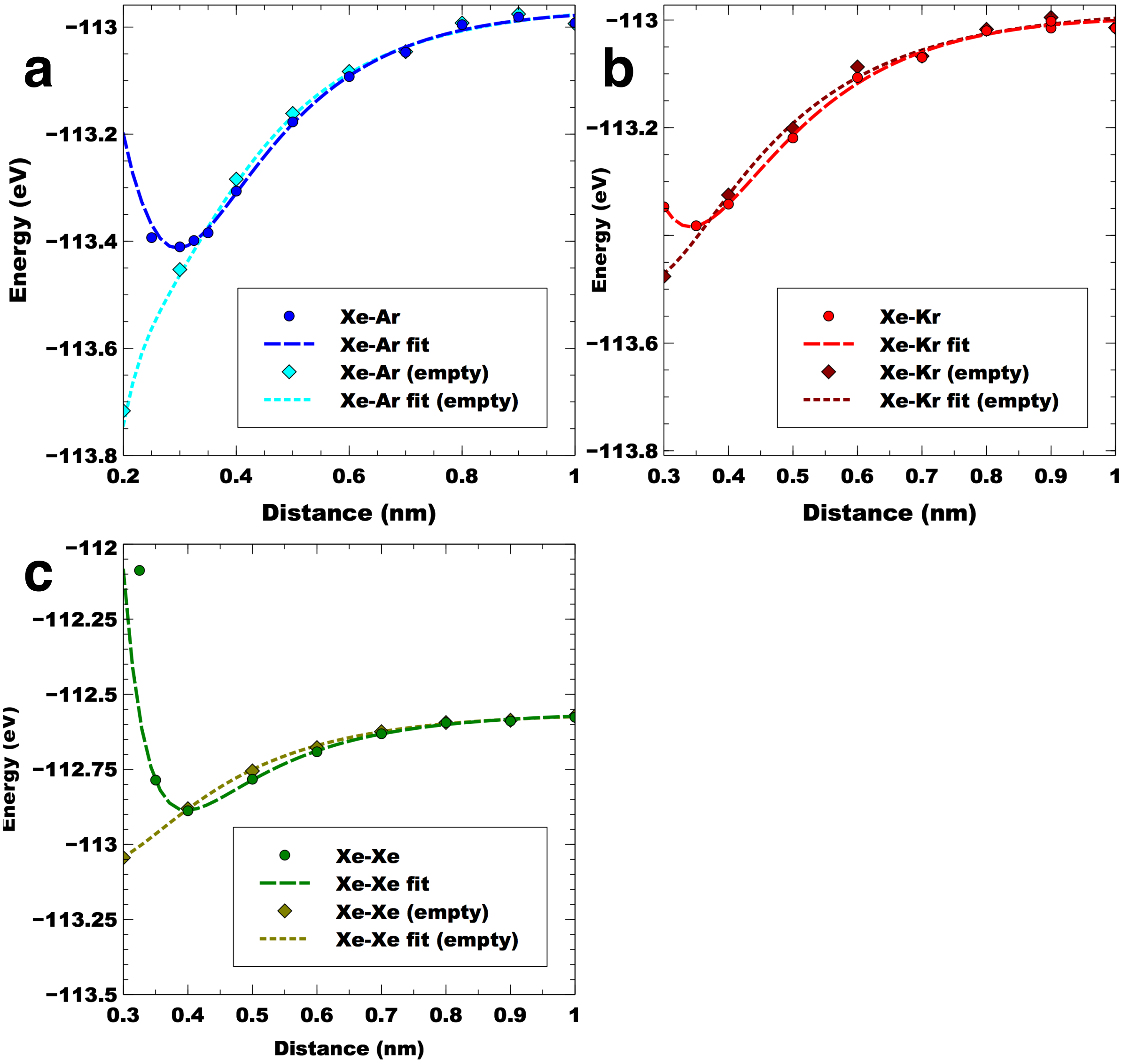

Supplementary Figure 7. Comparison of fits to calculated energies at different tip-surface separations. (**a**) Xe-Ar, (**b**) Xe-Kr, (**c**) Xe-Xe. The simulation curves shown in Fig. 4 in the main paper are produced as the difference between the fitted curves over occupied (Xe-X fit) and empty (Xe-X empty fit) sites. The curves were fitted with polynomial functions.

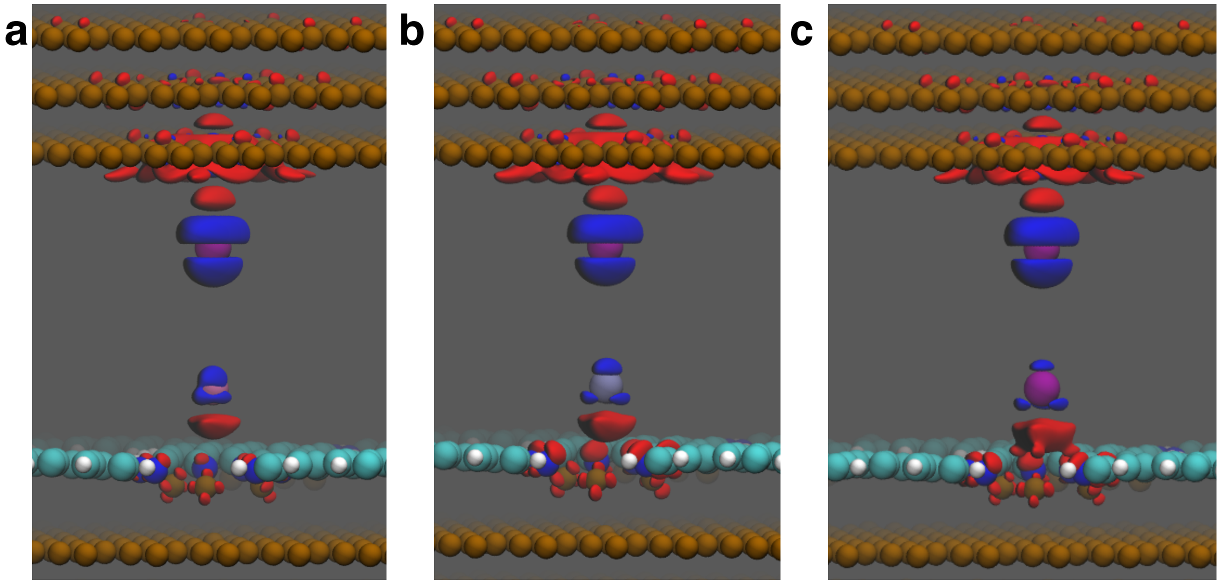

Supplementary Figure 8. **Comparison of induced differential electron density.** (**a**) Xe-Ar, (**b**) Xe-Kr, and (**c**) Xe-Xe. The red isosurface (+0.001 e) shows a gain in electron density and the blue isosurface (-0.0015 e) shows a loss. The induced differential density is calculated by subtracting from the full electron density of the tip/surface and rare gas atom system, the electron density of the isolated tip/surface system and the isolated rare gas atoms in exactly the same position as in the full system.

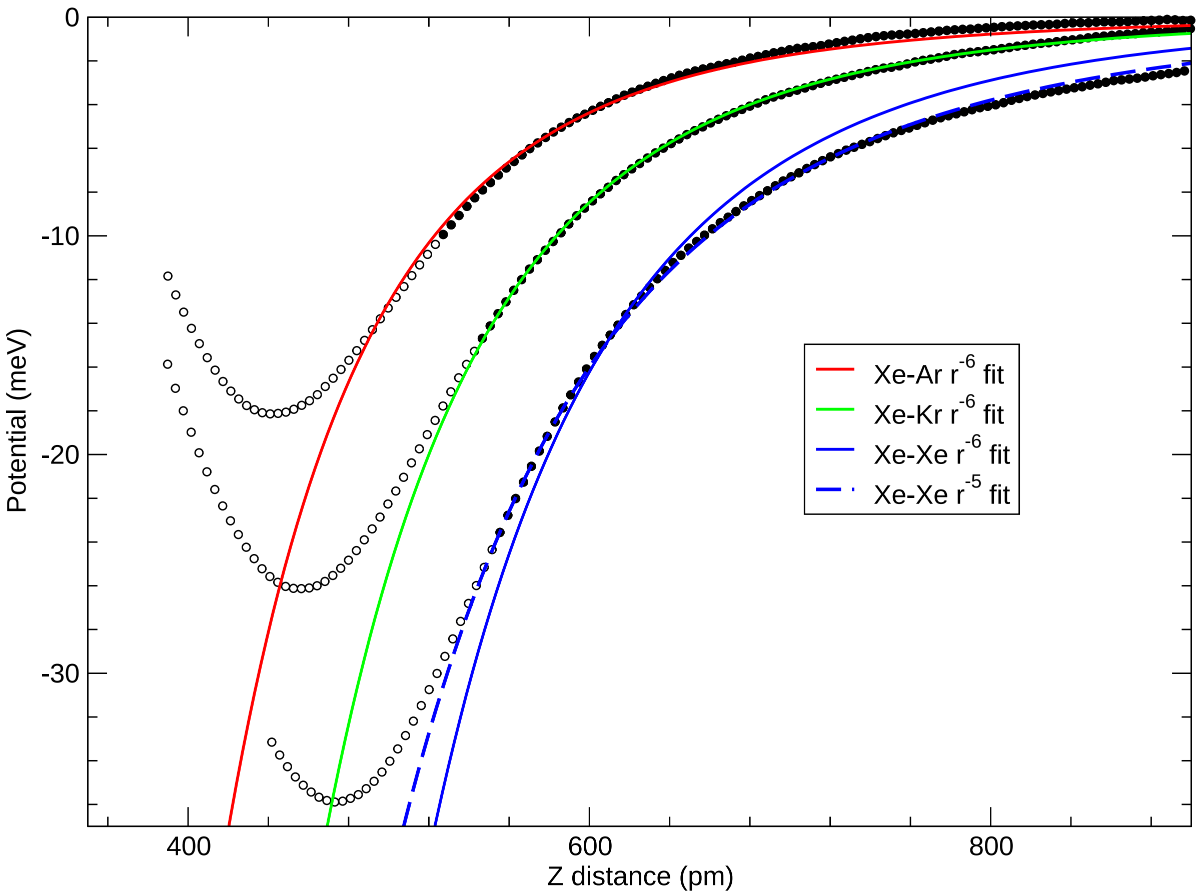

Supplementary Figure 9. **Fit of power laws to the experimental potential curves.** These one-parameter fits provide the C_6 coefficients given in Table I of the main paper. The full experimental curves are marked with open circles and the portion of the curve used for fitting by filled black circles. The positions of the experimental curves have been aligned to the DFT calculated minima by a shift of +38 pm, which results in an increase of the fitted C_6 coefficients by 7%. For the Xe-Xe curve, the optimal integer power r^{-5} is also shown for comparison. The discrepancy between the r^{-5} and the r^{-6} curves for Xe gives a good picture of the size of differences between fits to other powers of r for Ar and Kr. If a constant shift of the curves are simultaneously fitted, the curves will seem in markedly better agreement in the above figure, but this adjustment gives a very small ($< 1\%$) adjustment of the values of the C_6 values, so we prefer to use the minimal number of fitting parameters.

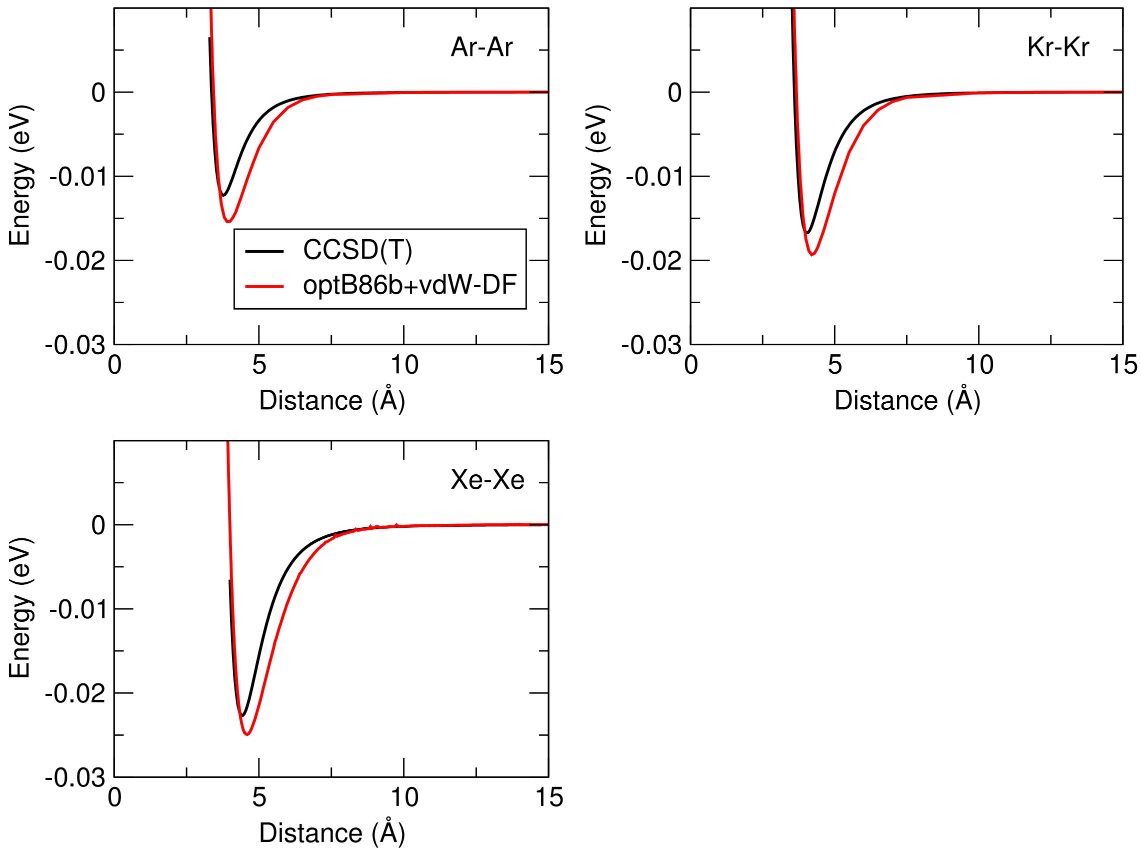

Supplementary Figure 10. **Benchmarking of the B86b functional.** Binding energy curves for Ar-Ar (top left), Kr-Kr (top right), Xe-Xe (bottom left). Shown are optB86b+vdW-DF curves and CCSD(T) reference data from Slavíček et al.[8].

Supplementary Table

Supplementary Table 1. Comparison of calculated adsorption energies of noble atoms in the various systems considered.

System	Noble atom	Adsorption energy (meV)
(111) Cu surface	Xe	-217<br>(Exp. -173 to -200, see [7] and references therein)
DPDI network	Ar	-140
DPDI network	Kr	-168
DPDI network	Xe	-213
DPDI network/(111) Cu	Ar	-82
DPDI network/(111) Cu	Kr	-162
DPDI network/(111) Cu	Xe	-179

SUPPLEMENTARY REFERENCES

[1] Park, J.-Y. *et al.* Modification of surface-state dispersion upon Xe adsorption: A scanning tunneling microscope study. *Phys Rev. B*, **62** R16341 (2000).

[2] König, T., Simon, G. H., Rieder, K.-H., Rust, H.-P. & Heyde, M. Superlattice structure of an Ar monolayer on Ag(111) observed by low-temperature scanning tunneling microscopy. *Phys Rev. B*, **78** 201407(R) (2008).

[3] Matena, M. *et al.* On-surface synthesis of a two-dimensional porous coordination network: Unraveling adsorbate interactions. *Phys Rev. B*, **90** 125408 (2014).

[4] Eigler, D. M., Lutz, C. P. & Rudge, W. E. An atomic switch realized with the scanning tunnelling microscope. *Nature* **352**, 600–603 (1991).

[5] Vasile, M. J., Grigg, D., Griffith, J. E., Fitzgerald, E., & Russell, P. E. Scanning probe tip geometry optimized for metrology by focused ion beam ion milling. *J. Vac. Sci. Technol. B*, **9** 3569-3572 (1991).

[6] Sun, X. & Yamauchi, Y. First-principles calculations of Xe-adsorbed Pd(111) and Cu(111) surfaces with an empirical correction of van der Waals interactions. *J. Appl. Phys.*, **110** 103701 (2011).

[7] Ruiz, V. G., Liu, W. & Tkatchenko, A. Density-functional theory with screened van der Waals interactions applied to atomic and molecular adsorbates on close-packed and non-close-packed surfaces. *Phys. Rev. B* **93**, 035118 (2016).

[8] Slavíček, P. *et al.* State-of-the-art correlated ab initio potential energy curves for heavy rare gas dimers: Ar_2,Kr_2, and Xe_2. *J. Chem. Phys.*, **119** 2102 (2003).
